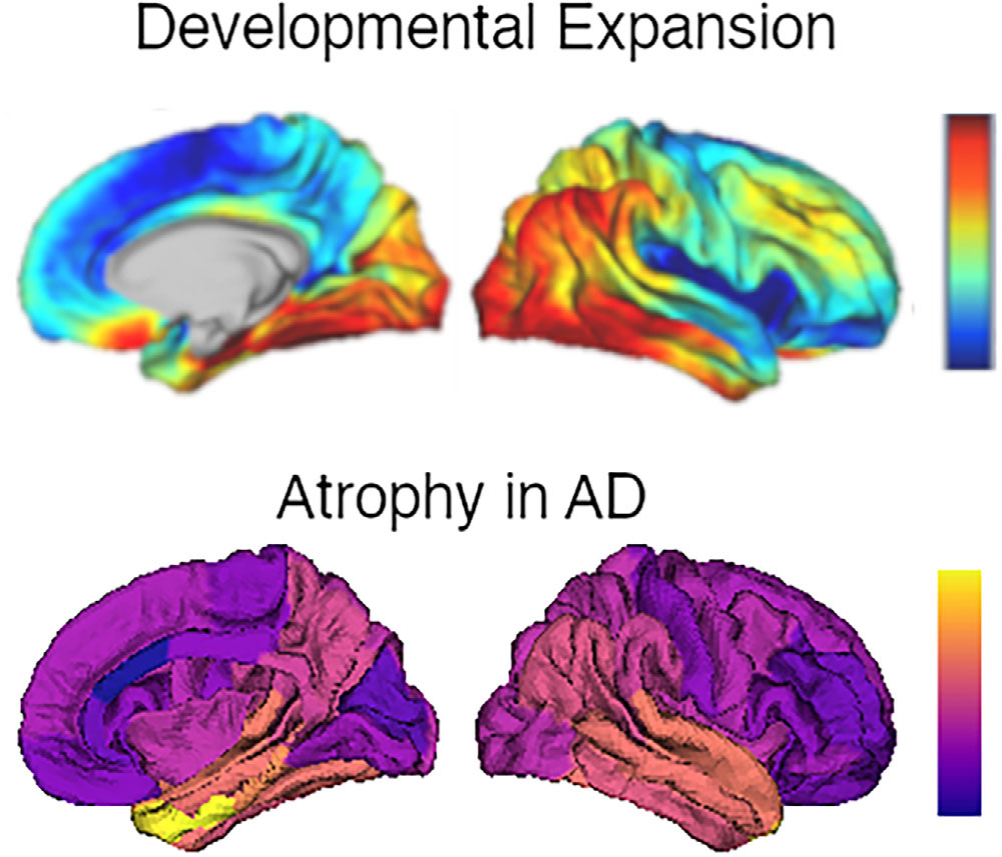# Imaging the impact of early and late life risk factors on the brain: a comparison

**DOI:** 10.1002/alz.094380

**Published:** 2025-01-09

**Authors:** Brian A. Gordon

**Affiliations:** ^1^ Washington University in St. Louis, Saint Louis, MO USA

## Abstract

**Background:**

Alzheimer disease (AD) is a chronic progressive neurodegenerative disorder that presents with cognitive dysfunction, memory loss, language difficulties, emotion dysregulation, and the eventual loss of motor function and death. Magnetic resonance imaging (MRI) shows early atrophy in the medial temporal lobes, which then spreads to the posterior temporal lobe, parietal lobe, and finally the frontal lobe with relative sparing of the sensorimotor cortex. Social disadvantage has been shown to have potentially additive impacts on aging trajectories. Over the first years of life the brain undergoes rapid expansion and maturation. Whereas sensory regions are relatively stable from birth, there is tremendous change in the temporal, parietal, and frontal regions which are responsible for higher order cognitive function. This change is paralleled by the development of motor, language, emotion regulation, and executive function. Early life events such as low birth weight, poverty, and adversity can impact neurodevelopment, including cortical expansion and connectivity. Given this mirroring between regional brain development and degeneration, researchers have hypothesized a “last in first out” mechanism underlying progressive neurodegeneration, but minimum direct comparisons have been performed. longitudinally studies older adults.

**Methods:**

This work brings together unique cohorts spanning the two ends of the lifespan. The Early Life Adversity, Biological Embedding (eLABE) study scans infants scanned longitudinally between birth and age three years of age. The Charles F. and Joann Knight ADRC and ADNI longitudinal follow middle and older‐aged individuals. Structural change is estimated a unique multimodal surface matching (MSM) pipeline that estimates the deformation of pairwise cortical surfaces to estimate cortical expansion or atrophy over time. Analyses will include all children with longitudinal MRI data as well as longitudinal MRI data to characterize healthy aging, individuals who convert to AD, as well as longitudinal change in within individuals with an AD diagnosis.

**Results:**

Preliminary results in each cohort (Figure 1) demonstrate highly similar spatial patterns between early life development and AD related cortical thinning.

**Conclusions:**

Although there has long been speculation that there is a reciprocity between regions that develop in childhood and those vulnerable to age and disease related decline, this work test this theory.